# Monolayer AsC_5_ as the Promising Hydrogen Storage Material for Clean Energy Applications

**DOI:** 10.3390/nano13091553

**Published:** 2023-05-05

**Authors:** Qiang Lu, Binyuan Zhang, Lianlian Zhang, Yulian Zhu, Weijiang Gong

**Affiliations:** College of Sciences, Northeastern University, Shenyang 110819, China

**Keywords:** AsC_5_ monolayer, hydrogen storage material, first-principles calculations

## Abstract

One of the critical techniques for developing hydrogen storage applications is the advanced research to build novel two-dimensional materials with significant capacity and effective reversibility. In this work, we perform first-principles unbiased structure search simulations to find a novel AsC${}_{5}$ monolayer with a variety of functionally advantageous characteristics. Based on theoretical simulations, the proposed AsC${}_{5}$ has been found to be energetically, dynamically, and thermally stable, supporting the viability of experiment. Since the coupling between H${}_{2}$ molecules and the AsC${}_{5}$ monolayer is quite weak due to physisorption, it is crucial to be enhanced by thoughtful material design. Hydrogen storage capacity can be greatly enhanced by decorating the AsC${}_{5}$ monolayer with Li atoms. Each Li atom on the AsC${}_{5}$ substrate is shown to be capable of adsorbing up to four H${}_{2}$ molecules with an advantageous average adsorption energy (E${}_{ad}$) of 0.19 eV/H${}_{2}$. The gravimetric density for hydrogen storage adsorption with 16Li and 64 H${}_{2}$ of a Li-decorated AsC${}_{5}$ monolayer is about 9.7 wt%, which is helpful for the possible application in hydrogen storage. It is discovered that the desorption temperature (T${}_{D}$) is much greater than the hydrogen critical point. Therefore, such crucial characteristics make AsC${}_{5}$-Li be a promising candidate for the experimental setup of hydrogen storage.

## 1. Introduction

As fossil fuel consumption increases and reserves decrease, it is imminent to develop new energy sources in today’s world [1,2]. Hydrogen energy is exactly this kind of secondary energy source that is green and low-carbon, widely used, and rich in sources [3,4,5]. The main links of the hydrogen energy industry chain include the preparation, storage, transportation, and utilization of hydrogen [6,7]. The hydrogen storage in the middle of the industry chain connects the production of hydrogen and the application of hydrogen. It is the key technology and prerequisite for the large-scale application of hydrogen. Realization of safe and effective hydrogen storage is the decisive factor for restricting the large-scale application of hydrogen energy [8,9,10]. The current technology for storing hydrogen in liquid and gaseous forms have restrictions in terms of weight, size, safety, and cost. A potential remedy for this issue, however, is the reversible storage of hydrogen in solid form [11,12,13,14,15,16]. The following two crucial considerations should be taken into account when selecting the hydrogen storage technique. The average adsorption energy (E${}_{ad}$) of adsorbed hydrogen on the possible adsorptive medium (−0.1 to −0.6 eV) is the first crucial factor [17]. The second is a gravimetric hydrogen storage capability that meets the U.S. Department of Energy’s (DOE) 2020 objective of 5.5 wt% [18]. Currently, we are unaware of any hydrogen storage materials that fulfill this stringent requirement. Hence, there is a critical need to develop a novel high-capacity material for storing hydrogen.

Among the various available options, carbonous materials possess a great potential for $H_{2}$ storage applications due to their large specific surface area, light weight, and numerous adsorption sites. After the successful exfoliation of graphene, the enthusiasm for developing inorganic carbon-based materials has grown to a great extent [19,20,21]. Due to their excellent electrical, optical, thermal, and mechanical properties, carbon-based materials are widely used [22,23]. Not only do they play an important role in nanoelectronic devices, but they also play a vital role in clean energy such as solar cells and hydrogen storage [24,25,26]. For instance, Ghanbari et al. investigated graphene’s potential application as a hydrogen storage material and found that graphene doped with Si and/or Ge has a large hydrogen molecule storage capacity [27]. Labrousse revealed that the volumetric and gravimetric capabilities of GeC${}_{3}$ may reach up to 98.41 g/dm${}^{3}$ and 7.25 wt%, respectively, [28]. Research into the storage of H${}_{2}$ in two-dimensional (2D) materials has shown that metal defects and decoration improve storage capacity relative to pure materials [29,30,31]. For example, H${}_{2}$ had only a limited interaction with pure MoS${}_{2}$ (adsorption energy of just −0.023 eV), but it was robustly adsorbed on the Ti-decorated MoS${}_{2}$ (−0.472 eV) [32]. At the same time, along with transition metals and alkali earth metals, alkali metal atoms are considered to be the preferred object because a large number of studies have confirmed that they play an important role in effectively improving hydrogen storage density due to their small cohesive energy and light atomic weight [33,34,35]. Lithium, as the alkali metal at the top of the periodic table, is preferred because of its light atomic weight and highest hydrogen storage weight ratio. For example, Li-doped graphene [36], CN monolayer [37], and B${}_{2}$S monolayer [38] all show excellent hydrogen storage performance.

**Figure 1:**
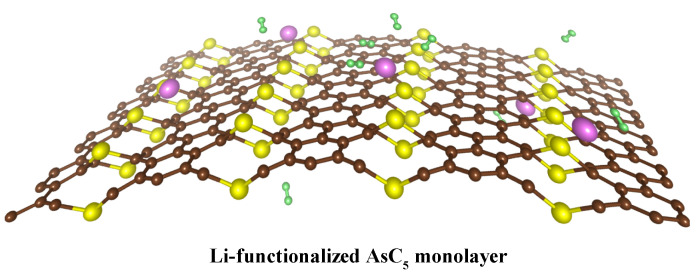
Schematic diagram of hydrogen storage on Li-functionalized As$C_{5}$. The green dots denote the H atoms, yellow dots describe the As atoms, brown dots correspond to C atoms, and purple dots are the Li atoms.

This research is inspired by the aforementioned reasons; thus, given that C materials have outstanding conduction and stability qualities, our main focus is on AsC${}_{5}$ compositions rich in C. We conduct a thorough structural search to identify the monolayer that is the most stable [39]. As anticipated, we observe a novel AsC${}_{5}$ monolayer with a graphene-like buckling structure (see Figure 1). Following that, phonon calculations and AIMD simulations are used to examine the structural stability. Then, density functional theory (DFT) simulations are used to examine the viability of the Li-decorated AsC${}_{5}$ film as a hydrogen storage material. We confirm that the hydrogen binding energy is enhanced by Li decoration on the AsC${}_{5}$ monolayer. Moreover, it is found that in the fully loaded case, the hydrogen storage density is up to 9.7 wt%, which is in excellent agreement with the standards set by the US Department of Energy. Additionally, the desorption temperature (T${}_{D}$) of this system ranges from 243 to 357 K, significantly greater than hydrogen’s critical temperature (33 K). In this regard, AsC${}_{5}$-based materials are considered to be candidates for excellent hydrogen storage of clean energy applications.

## 2. Method

To identify the lowest energy structures of AsC${}_{5}$ monolayers, the particle swarm optimization (PSO) approach is used inside the evolutionary process as implemented in the CALYPSO package [39,40]. A total of 60% of the structures from the first generation with lower Gibbs free energies are chosen for PSO construction of the structures of the following generation. In the next generation, 40% of the structures are produced at random. The variety of the structures is greatly increased by these processes, which is critical for the efficiency of a structural global search. Significantly, theoretical computations are crucial for both finding novel 2D materials and comprehending the underlying physical processes behind observed experimental findings [41,42,43,44].

The Vienna ab initio Simulation Package (VASP) is employed for our first-principles computations [45,46]. In this code, the Kohn–Sham equations are solved within a pseudopotential approximation, in which the popular generalized gradient approximation (GGA) of the PBE function is utilized to describe the exchange-correlation energy and potential between electrons [47]. Furthermore, van der Waals corrections have been included using the DFT-D3 method of Grimme to model the interaction at the interface [48,49]. For the structural relaxation, the total energy tolerances and maximum atomic forces are within $1.0\times 10^{-6}$ eV and 0.01 eV/Å, respectively. The isolation of slab models are created with a vacuum of 20 Å to avoid unphysical interactions between periodically repeated images. A mesh of 8×4×1 *k* for the unit cell and 4×2×1 *k* for the 2×2 supercell system are used [50] for the integration of the Brillouin zone, and the kinetic energy cutoff is set to 500 eV. The ab initio Molecular Dynamic Simulation (AIMD) is utilized to predict thermal stability, and a Nose–Hoover thermostat framework is used for temperature regulation [51].

## 3. Results and Discussion

**Figure 2:**
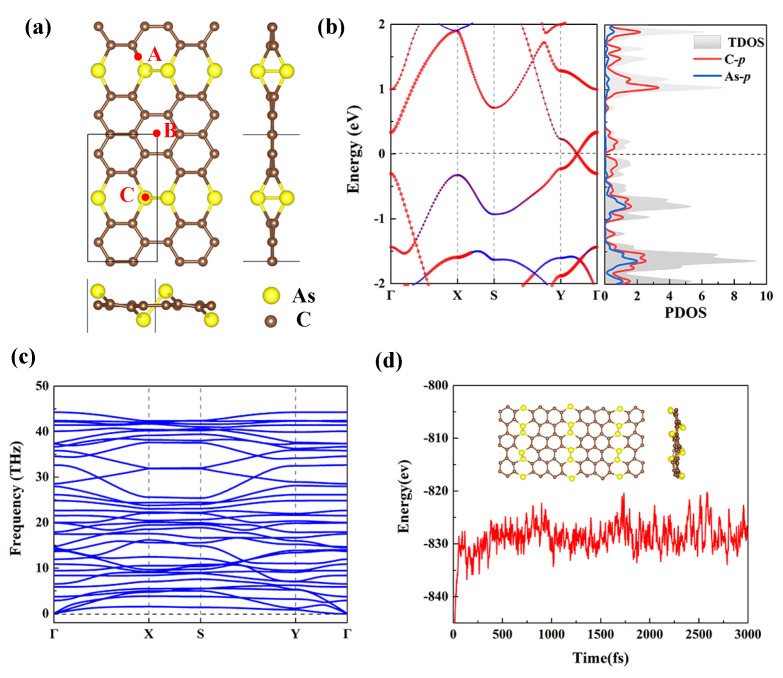
(**a**) Optimized crystal structures of the AsC${}_{5}$ monolayer (Top and side views). (**b**) The electronic band structure and PDOS spectra of the AsC${}_{5}$ monolayer. (**c**) Phonon dispersion curves of AsC${}_{5}$ monolayer. (**d**) AIMD plots at 1000 K for 3 ps of AsC${}_{5}$. The inset shows that the structure of AsC${}_{5}$ after dynamics simulations.

According to the PSO technique and DFT calculations, we design a novel AsC${}_{5}$ monolayer, as shown in Figure 2a. One can find that ten C and two As atoms construct the AsC${}_{5}$ monolayer in the rectangle-shaped unity cell, and the AsC${}_{5}$ monolayer displays the buckled structure. Next, relaxed lattice constants and monolayer thicknesses of AsC${}_{5}$ are found to be a=4.41 Å, b=8.08 Å, and 1.83 Å. The C-C and As-C bond lengths are calculated as 1.42 Å and 1.98 Å, respectively. Furthermore, Figure 2b clearly shows the electronic band structure and projected density of states (PDOS) of AsC${}_{5}$. It is shown that the AsC${}_{5}$ monolayer exhibits a similar Dirac cone with a tiny band gap (0.01 eV). From the PDOS, we observe that the p-orbits of C atoms contribute mostly to the conduction band minimum and valence band maximum of the AsC${}_{5}$ monolayer.

The structural stability of any material is critical for its practical application; thus, we estimate the phonon dispersion curve to prove dynamical stability of AsC${}_{5}$. From Figure 2c, one can see that no negative frequencies are identified in any of the acoustic branches which branch out from Γ. This strongly suggests that the AsC${}_{5}$ monolayer is dynamically stable. In addition, the thermodynamic stability of AsC${}_{5}$ monolayer is confirmed by employing AIMD simulations, and we find no significant reorganization of structure and bonds broken after the relaxation of 3 ps at 1000 K with energies fluctuating slightly (see Figure 2d). Finally, the cohesive energy of AsC${}_{5}$ is calculated to explore the mechanical strength. According to the research results, the AsC${}_{5}$ monolayer has the cohesive energy of 6.72 eV/atom, which is higher than that of arsenene (2.95 eV/atom) [52] and almost as high as that of graphene (7.98 eV/atom) [53]. Based on the above data, we expect that the AsC${}_{5}$ monolayer can be synthesized experimentally.

**Figure 3:**
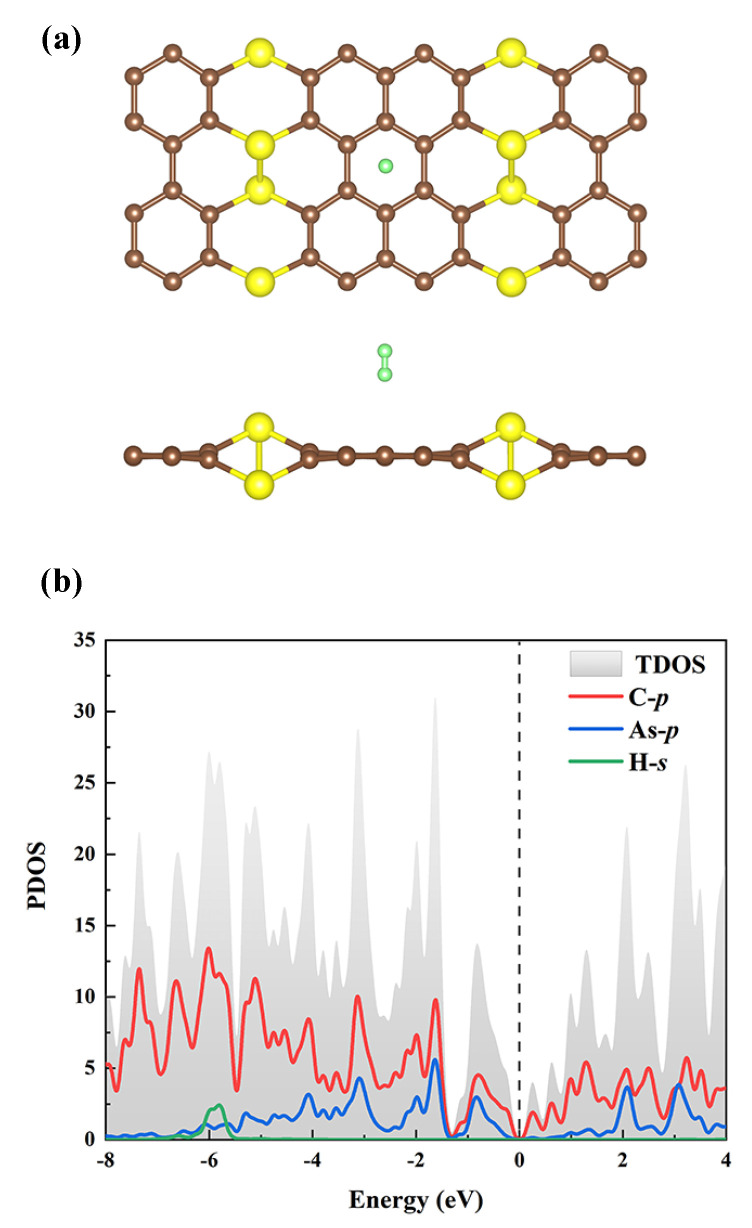
(**a**) Optimization diagram of the adsorption of H${}_{2}$ on the AsC${}_{5}$ monolayer. (**b**) PDOS of single H${}_{2}$ adsorbed on pure AsC${}_{5}$ at the B adsorption site.

To evaluate the hydrogen storage capability of pristine AsC${}_{5}$ nanosheets, we adopt a 2×2×1 supercell and put one H${}_{2}$ molecule on various potential locations to comprehensively investigate H${}_{2}$ adsorption capability on the AsC${}_{5}$ monolayer. As shown in Figure 2a, three potential adsorption sites are selected depending on the symmetry of AsC${}_{5}$, including the bridge site (A) of the C-As bond, the hollow site (B) above the C-C ring, and the top site (C) on the As atom. The average adsorption energy ($E_{ad}$) is calculated by the following expression:

(1) $$\begin{array}{c} E_{ad}=\left(E_{AsC_{5}+nH_{2}}-E_{AsC_{5}}-nE_{H_{2}}\right)/n, \end{array}$$

where $E_{AsC_{5}+nH_{2}}$ is the energy of the AsC${}_{5}$ monolayer after adsorption of *n* H${}_{2}$ molecules and $E_{AsC_{5}}$ is the energy of the pristine AsC${}_{5}$ monolayer. $E_{H_{2}}$ represents the energy of single H${}_{2}$ molecule. The calculated $E_{ad}$ of a H${}_{2}$ molecule at A-site, B-site, and C-site is −0.037 eV, −0.043 eV, and −0.020 eV per H${}_{2}$, respectively (Table 1). The vertical adsorption distances of H${}_{2}$ to AsC${}_{5}$ are 2.30 Å, 1.71 Å, and 2.52 Å, respectively. This is caused by the buckling structure of the AsC${}_{5}$ material. As C-site is close to the protruding As atom, it receives strong repulsion, so the vertical adsorption distance is larger and the corresponding adsorption energy is reduced. Adsorbed H${}_{2}$ is found to have a H-H bond length (l) of 0.75 Å, which is equal to the free H${}_{2}$, indicating that the adsorbed H${}_{2}$ is unaffected by AsC${}_{5}$. Furthermore, it is discovered that the minimal H${}_{2}$ adsorption energy occurs at the B-site ( see Figure 3a), and its adsorption energy, which is far below −0.1 eV, is insufficient to store hydrogen. The electrons in the s shell of the H${}_{2}$ and the p shells of carbon are hybridized, with the energy close to −6.0 eV, according to the comparison of the PDOS depicted in Figure 2b and Figure 3b.

**Figure 4:**
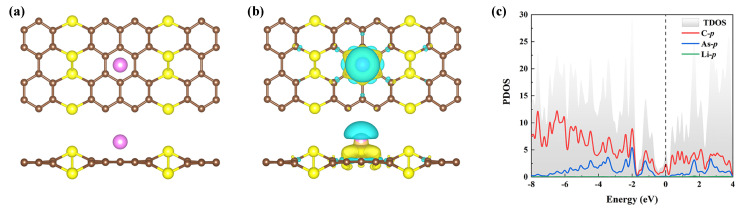
(**a**) Optimized structure and (**b**) charge density differences of Li-decorated AsC${}_{5}$ on the B-site. The isosurface is set to 0.002 e/Bohr${}^{3}$. (**c**) The corresponding PDOS of the AsC${}_{5}$ monolayer decorated with one Li atom.

In order to study the effect of Li modification on the hydrogen storage capacity of AsC${}_{5}$, we start by putting a single Li atom at each of the adsorption sites depicted in Figure 2a. The decorated Li atom’s binding energy (E${}_{b}$) to the AsC${}_{5}$ is given by the equation below:

(2) $$\begin{array}{c} E_{b}=\left(E_{AsC_{5}-mLi}-E_{AsC_{5}}-mE_{Li}\right)/m, \end{array}$$

where E${}_{AsC_{5}-mLi}$, E${}_{Li}$, and *m* represent the total energy of the AsC${}_{5}$ compound with *m* doped Li atoms, the energy of a free Li atom, and the number of Li atoms adsorbed, respectively. Table 1 summarizes the binding energy characteristics of Li and the corresponding charge transfer at the position considered on the surface of AsC${}_{5}$. The findings reveal that the Li atoms have a preference for being adsorbed above the center site of the hexagonal honeycomb (B site), with an E${}_{b}$ of −2.36 eV (see Figure 4a for the optimized structure). The decorating of the Li atom on the AsC${}_{5}$ is an exothermic process since the E${}_{b}$ value is negative. Additionally, it is important to note that the absolute value of the E${}_{b}$ of the Li atom’s adsorption on B-site is larger than the cohesive energy of Li bulk (1.61 eV). This demonstrates the potential dispersion and stability of Li atom on the AsC${}_{5}$ [54].

Next, we investigate the charge density difference between Li-adsorbed AsC${}_{5}$ and pristine AsC${}_{5}$ to explore the electrochemical contact between Li and AsC${}_{5}$ with Equation (2):

(3) $$\begin{array}{c} \Delta \rho =\rho_{AsC_{5}-Li}-\rho_{AsC_{5}}-\rho_{Li}. \end{array}$$

$\rho_{Li},\rho_{AsC_{5}}$, and $\rho_{AsC_{5}-Li}$ are the charge densities of an isolated Li, pristine AsC${}_{5}$, and adsorption systems, respectively. Figure 4b shows an obvious charge redistribution in the interface area, where charge accumulation is primarily on the AsC${}_{5}$ surface and depletion is near the Li atoms. This suggests that Li ions bond strongly to the AsC${}_{5}$ monolayer as a consequence of electrons being transported from Li to the substrate. Additionally, the precise quantity of the charge transfer is determined by Bader charge analysis (see Table 1). In the stable adsorption sites, around 0.57–0.77 e is transported from Li to AsC${}_{5}$ sheet, indicative of an ionic interaction between Li and AsC${}_{5}$. To understand the conductivity of sodiated AsC${}_{5}$, the PDOS is calculated and shown in Figure 4c. It is worth noting that when Li is adsorbed on the AsC${}_{5}$ monolayer, the system exhibits metallicity due to a slight downward shift of the conduction band compared with the pristine AsC${}_{5}$.

After assessing the viability of Li decoration on the AsC${}_{5}$ monolayer, further investigation into the hydrogen storage capability of this system can be performed. The consecutive adsorption energy (E${}_{ca}$) of H${}_{2}$ molecules can be calculated as follows:

(4) $$\begin{array}{c} E_{ca}=E_{AsC_{5}-Li+\left(n-1\right)H_{2}}+E_{H_{2}}-E_{AsC_{5}-Li+\left(n\right)H_{2}}. \end{array}$$

**Figure 5:**
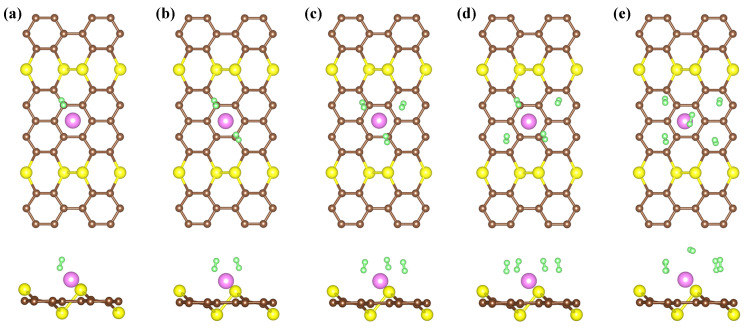
The optimized structures of 1–5 (**a**–**e**) H${}_{2}$ adsorbed on AsC${}_{5}$-Li.

In this formula, *n* stands for the number of H${}_{2}$ molecules, and E${}_{AsC_{5}-Li+\left(n-1\right)H_{2}}$ and E${}_{AsC_{5}-Li+\left(n\right)H_{2}}$ are the total energies of *n*H${}_{2}$ molecules and (n−1)H${}_{2}$ absorbed on the AsC${}_{5}$ monolayer of the decorated system by Li, respectively. It can be seen from Figure 5 that the adsorption of hydrogen steadily rises from left to right. The substrate AsC${}_{5}$-Li shows slight deformation following hydrogen adsorption, indicating that the adsorbent has excellent cycle performance. The results show that the first H${}_{2}$ molecule is adsorbed on the AsC${}_{5}$-Li compound with an adsorption energy of −0.31 eV/H${}_{2}$ and the bond length of H-H is 0.76 Å (Table 2). It should be noticed that the E${}_{ad}$ of H${}_{2}$ on AsC${}_{5}$-Li is nearly eight times greater than that on the pure AsC${}_{5}$ monolayer. As more H${}_{2}$ molecules are introduced to the compound, the E${}_{ad}$ value of the molecules decreases until it reaches −0.24 eV/H${}_{2}$ for the AsC${}_{5}$-Li compound. The adsorption energy, which is suggested to be in the region of −0.1 to −0.6 eV, is obviously perfect for hydrogen storage.

**Figure 6:**
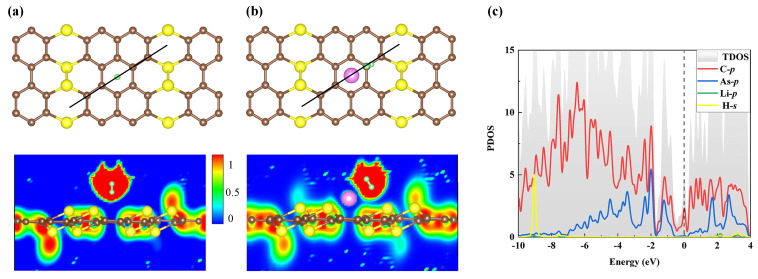
ELF profiles of the adsorption of one H${}_{2}$ on (**a**) the pure AsC${}_{5}$ or (**b**) the Li-decorated one. (**c**) The corresponding PDOS spectra.

At the same time, electron localization function (ELF) is investigated to further study the interaction between H${}_{2}$ and pure AsC${}_{5}$ or Li-modified AsC${}_{5}$, as shown in Figure 6. The blue areas indicate that the ELF value between H${}_{2}$ and pure AsC${}_{5}$ is very close to zero (see Figure 6a), meaning that no electrons are shared in this adsorbed system and no new bonds are formed. This is strong evidence that H${}_{2}$ can only physically adsorb to the pure AsC${}_{5}$ monolayer, since no ELF region of chemical bonding has been observed. On the other hand, a significant electron localization overlap is detected between adsorbed H${}_{2}$ and AsC${}_{5}$-Li, as shown in Figure 6b. One can find the electron sharing in the AsC${}_{5}$-Li mode and the chemical properties of H${}_{2}$ adsorption by Li-decorated AsC${}_{5}$. Next, Figure 6c shows that the prominent peak of H-s around 9 eV is overlapped strongly with the Li-p, the C-p, and As-p. This illustrates the strong hybridization between the adsorbed H${}_{2}$ and the decorated Li atom in the modified AsC${}_{5}$. Additionally, the PDOS analysis combined with the total electron density and ELF results confirms the strong adsorption of H${}_{2}$ on AsC${}_{5}$-Li.

**Figure 7:**
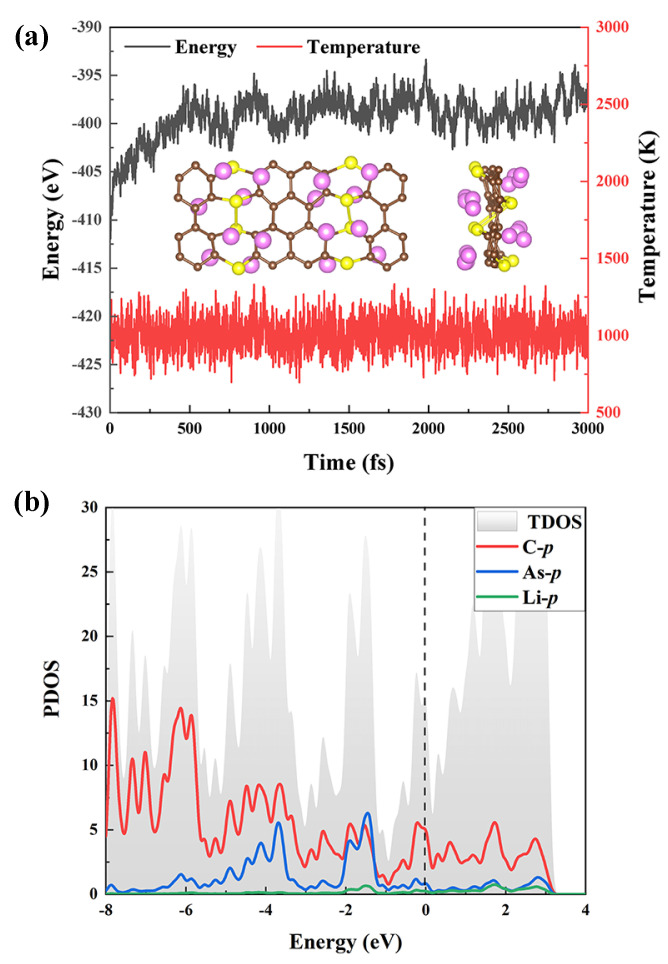
(**a**) Energy and temperature fluctuations during AIMD simulation. Inset: the configurations after 3 ps at 1000 K. (**b**) The PDOS spectra of AsC${}_{5}$-16Li.

The possibility of adding more Li atoms to each side of the AsC${}_{5}$ monolayer is also taken into account. The calculated average E${}_{b}$ of 4, 8, and 16 Li atoms decorated on the AsC${}_{5}$ monolayer are −0.44, −0.41, and −0.36 eV/Li, respectively. To confirm the stability of AsC${}_{5}$-xLi, we perform the AIMD simulation of AsC${}_{5}$-16Li at 1000 K for 3 ps. As shown in Figure 7a, AsC${}_{5}$-16Li could keep its structural integrity without significant alterations. Furthermore, in the AIMD simulation, there is a tiny variation in energy. All of the findings suggest that AsC${}_{5}$-16Li is thermally stable at 1000 K. As can be seen in Figure 7b, we also carry out an analysis of the PDOS results. They confirm the strong bond between the AsC${}_{5}$ monolayer and Li atom by demonstrating an evident hybridization between the p-orbits of the C and As atoms and the p-orbits of the Li atoms. Moreover, as mentioned above, AsC${}_{5}$ has a tiny band gap (0.01 eV) and presents semi-metallic properties, indicating good conductivity, which has a positive influence on Li embedding/desorption.

Regarding practical H${}_{2}$ storage applications, reversible H${}_{2}$ adsorption/liberation at room temperature is crucial. The temperature of desorption ($T_{D}$) typically increases with increasing adsorption energy for H${}_{2}$ molecules. $T_{D}$ for H${}_{2}$ molecules with different adsorption numbers at AsC${}_{5}$-16Li has been calculated in the current investigation using the equation below [55]:

(5) $$\begin{array}{c} T_{D}\left(K\right)=\left(\frac{|E_{b}|}{K_{B}}\right){\left(\frac{\Delta S}{R}-\ln P\right)}^{-1}. \end{array}$$

**Figure 8:**
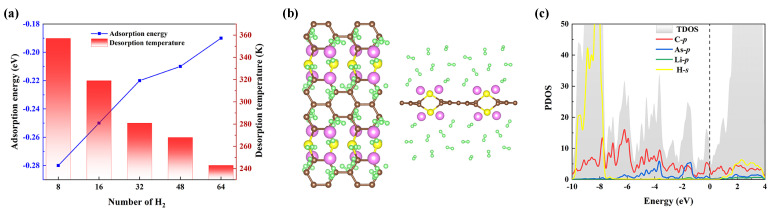
(**a**) Average adsorption energies and desorption temperature as a function of the number of adsorbed hydrogen molecules on AsC${}_{5}$-16Li. (**b**) The optimized systems of maximum hydrogen molecules adsorption on AsC${}_{5}$-16Li. (**c**) PDOS of 64 H${}_{2}$ adsorbed on AsC${}_{5}$-16Li.

K${}_{B}$, *R*, and ΔS are, respectively, the Boltzmann constant, gas constant (8.31 J/mol K), and change in the H${}_{2}$ molecule’s entropy (S) from gas to liquid phase at the balance pressure (i.e., the 1 atm). The gravimetric density of H${}_{2}$ storage is calculated by employing the formula

(6) $$\begin{array}{c} C_{g}=\frac{M_{H_{2}}}{M_{H_{2}}+M_{host}}, \end{array}$$

where $M_{H_{2}}$ and $M_{host}$ denote the total weight of the adsorbed H${}_{2}$ and the Li-decorated AsC${}_{5}$, respectively. For the decorated systems (AsC${}_{5}$-16Li), we discover that the $T_{D}$ values lie in the range of 243–357 K (see Figure 8a), which are significantly larger than most other 2D materials such as Be${}_{2}$C-Li/K (163/129 K), BP-Li/Na (116/95 K), and GeC${}_{3}$ (224 K). We also note that the $T_{D}$ of the AsC${}_{5}$-16Li substrate is significantly greater (>7 times) than the hydrogen critical temperature.

As shown in Figure 8b, on the AsC${}_{5}$-16Li substance, a maximum of 4 H${}_{2}$ molecules can be adsorbed on each Li with an energy of −0.19 eV/H${}_{2}$, so the gravimetric density of hydrogen storage of this system is enhanced to 9.7 wt%. A comparison of the s-orbit of H and p-orbit of Li in the PDOS is given in Figure 8c, and the results demonstrate no clear hybridization, implying their weak interaction with each other. It is known that the hydrogen storage of carbon-based materials has been the subject of numerous prior research. The results in Table 3 show that the hydrogen density varies greatly for different materials, depending on the choice of the doped metal atoms and substrate. Note also that the hydrogen storage capacity found in our research is greater than that of the majority of the materials mentioned. Furthermore, by taking into account the outstanding thermodynamically stable conditions, which is another crucial criterion for hydrogen storage materials, one can ascertain that Li-decorated AsC${}_{5}$ is a viable candidate for the application of the hydrogen storage material.

## 4. Summary

To summarize, in this work we have employed the first-principles DFT computations to establish and calculate the pristine and Li-decorated AsC${}_{5}$ sheets. As a result, it has been found that Li atoms on the surface of the AsC${}_{5}$ monolayer could be adjusted in a stable manner with their negative adsorption energy. The hydrogen molecule has a modest interaction with unmodified AsC${}_{5}$, but a strong interaction with AsC${}_{5}$ that is decorated with a single Li atom. H${}_{2}$ adsorbed on the modified AsC${}_{5}$ has an adsorption energy of −0.31 eV, which is nearly 7 times greater than that of the pristine AsC${}_{5}$ (−0.04 eV). Overall, up to 64 H${}_{2}$ molecules could be adsorbed on both sides of AsC${}_{5}$-16Li, and the average energy is well within the ideal range for desorption temperature and effective hydrogen storage properties. The maximal gravimetric hydrogen storage capacity of Li-decorated AsC${}_{5}$ can reach 9.7 wt%, which is greater than that of the majority of carbon-based materials. All of these appealing properties indicate that the Li-doped AsC${}_{5}$-based material has great potential as a reversible hydrogen storage material that can be used as clean alternative fuel for transportation.

## Figures and Tables

**Table 1:** The calculated adsorption energy (E${}_{ad}$), the adsorption height (h), H-H bond length (l) of hydrogen molecule, and the calculated binding energy (E${}_{b}$) of a single Li-decorated AsC${}_{5}$ system and Bader charge analysis of a Li atom at A, B, and C-sites.

	E${}_{ad}$ ( eV)	h (Å)	l (Å)	E${}_{b}$ ( eV)	Q
A	−0.037	2.30	0.75	−2.33	0.57
B	−0.043	1.71	0.75	−2.36	0.77
C	−0.020	2.52	0.75	−2.34	0.63

**Table 2:** Calculated average Li-H${}_{2}$ distance (d), the average H-H bond lengths (l), the average adsorption (E${}_{ad}$), and the consecutive adsorption (E${}_{ca}$) energies of H${}_{2}$ molecules.

n	d (Å)	l (Å)	E${}_{ad}$	E${}_{ca}$
1	2.04	0.76	−0.31	0.31
2	2.07	0.76	−0.29	0.29
3	2.49	0.76	−0.28	0.24
4	2.43	0.76	−0.27	0.23
5	2.70	0.75	−0.24	0.12

**Table 3:** Hydrogen density of different materials.

Structure	wt%
B@r57-Li4	10
Li-decorated T4,4,4-graphyne	10.46
Ti-decorated MoS${}_{2}$	5.93
Pd-decorated Si${}_{2}$BN	6
Ti-decorated graphene	7.8
GeC${}_{3}$	7.25
Li-decorated Be${}_{2}$C	10.21
Li-decorated AsC${}_{5}$ (This work)	9.7

## Data Availability

Data available on request from the authors.
